# A novel loss-of-function mutation in *MCMDC2* is associated with male infertility

**DOI:** 10.1038/s41439-026-00342-6

**Published:** 2026-03-12

**Authors:** Ori Cohen, Florence Abou, Netanel Waldenberg, Moran Gershoni, Sandra E. Kleiman, Ruti Parvari, Shlomi Barak

**Affiliations:** 1 Ben-Gurion University of the Negev, National institution of Biotechnology in the Negev, Be’er Sheva, Israel; 2 Ben-Gurion University of the Negev, The Shraga Segal Department of Microbiology, Immunology and Genetics Faculty of Health Sciences, Be’er Sheva, Israel; 3 The Multidisciplinary Center for Male and Female Reproduction and Fertility, Male Infertility, Tel Aviv, Israel; 4 Institute of Animal Sciences, Agriculture Research Organisation, Volcani Center, Rishon LeZion, Israel; 5 Male Fertility Clinic and Sperm Bank, Lis Maternity Hospital, Tel Aviv Sourasky Medical Center, affiliated with the Sackler Faculty of Medicine, Tel Aviv, Israel; 6 Assuta Ashdod University Hospital, Reproductive Service, Ashdod, Israel

**Keywords:** Genetics research, Genetics

We report a homozygous loss-of-function mutation in the *MCMDC2* gene ((NM_173518.5): c.255_260delinsGG; p.Leu86ValfsTer12) in a patient with non-obstructive azoospermia due to maturation arrest. *MCMDC2* is important in meiotic recombination and DNA repair during spermatogenesis. Previously reported mutations in *MCMDC2* are associated with similar phenotypes. These findings expand the gene’s mutational spectrum and support its role in the genetic etiology of male infertility.

Infertility is defined as the inability to conceive for 12 months or more despite having intercourse without contraception^1^. Approximately 15% of couples worldwide suffer from infertility, with 50% of cases being due to male infertility^1,2^. About 20% of cases of male infertility are idiopathic, and some are believed to be caused by genetic defects^3^. Azoospermia is defined as the total absence of spermatozoa in the ejaculate, reported to be present in about 15% of men undergoing fertility investigation^2^. Non-obstructive azoospermia (NOA) associated with spermatogenic failure is considered to be a challenging form of azoospermia, most commonly managed by surgical sperm retrieval followed by intracytoplasmic sperm injection (ICSI)^4^. Many genes have already been identified as causative factors for NOA in humans, highlighting the importance of genetic investigation in affected individuals^5^.

This study reports a novel variant in the *MCMDC2* gene associated with male infertility. A 28-year-old Ashkenazi Jewish male presented with primary infertility after attempting to conceive for 3 years with no success. Semen analysis performed 2 years earlier revealed normal ejaculate volume and pH, as well as azoospermia. Repeat semen analyses remained unchanged. His past medical history was unremarkable. On physical examination, the patient had a normal body mass index. The testes were firm, nontender and of low volume (8–9 ml) bilaterally, with no palpable masses. The spermatic cord structures were normal. No varicoceles were noted on either side. A reproductive endocrinology evaluation of his current presentation showed normal levels of follicle-stimulating hormone (FSH) and luteinizing hormone (LH), but low testosterone (Table 1). His karyotype was normal, and no Y-chromosome microdeletions were detected. Microdissection testicular sperm extraction revealed no spermatozoa in the examined tissue. Histopathological analysis demonstrated complete maturation arrest.

**Table 1:** Genotype–phenotype correlation in *MCMDC2*-related male infertility: Our patient (II-6 in this case) compared with published cases*.

Patient’s name (Reference)	II-6 in this case	M1 (17)	M2 (17)	M3 (17)	M4 (17)	P0085 (18)	Participant no. 32 (11)	Participant no. 33 (11)
Age	29	28	37	27	34	N/A	30	32
Testis histology	Maturation arrest	Hypospermatogenesis	Meiotic arrest	Meiotic arrest	Meiotic arrest	Spermatogenic arrest: meiotic (MeA)	Maturation arrest	N/A
cDNA alteration	c.255_260delinsGG	c.94G>T	c.1360G>T	c.1956G>T	c.685C>T	c.1795C>T	c.1363C>T c.1767T>A	c.1767T>A
Variant allele	Homozygous	Homozygous	Homozygous	Homozygous	Homozygous	Homozygous	Heterozygous Heterozygous	Homozygous
Protein alteration	p.Leu86ValfsTer12	p.Asp32Tyr	p.Val454Phe	p.Arg652Ser	p.Gln229Ter	p.Arg599Ter	p.Gln455Ter p.Tyr589Ter	p.Tyr589Ter
Variant type	Frameshift	Missense	Missense	Missense	Nonsense	Nonsense	Nonsense Nonsense	Nonsense
FSH (IU/l)	6.1	3.59	24.1	6.71	19.25	N/A	10.5	33.7
LH (IU/l)	6	3.83	19.8	3.8	8.34	N/A	8.1	21.8
Testosterone (nmol/l)	8.5	15.08	48.887	16.608	23.750	N/A	9.9	6.0
Allele frequency in the human population (all individuals in gnomAD)	N/A	0.00002816	0.000008038	N/A	N/A	0.0001098	0.00001069 0.0004123	0.0004123
Testicular volume (Left; ml)	8–9	15	12	12	10	5–10	20	15
Testicular volume (Right; ml)	8–9	15	12	12	12	5–10	20	12

^*^All patients exhibited a normal male karyotype (46,XY) without evidence of Y-chromosome microdeletions. None had a notable personal medical history, a family history of infertility or related disorders. *N/A* information not available.

Whole-exome sequencing (WES) was performed on genomic DNA extracted from peripheral blood. Data were analyzed using the ‘Franklin by Genoox’ platform (https://franklin.genoox.com/). Variant filtering focused on rare homozygous single-nucleotide variants and small insertion–deletions, with population allele frequency <2%, and on exonic deletions detected from WES coverage-based copy number analysis. Our analysis focused on variants within a custom gene panel associated with human male infertility, curated from the literature and genomic databases, including OMIM (https://www.omim.org/), the Monarch Initiative (https://monarchinitiative.org/), the Mouse Genome Informatics database (MGI: https://www.informatics.jax.org/) and Genecards (https://www.genecards.org/). Gene selection was also informed by previously published reviews of monogenic causes of male infertility^6–8^. In addition, we constructed a secondary panel comprising candidate genes previously suggested to play roles in male reproductive function, although causality in human fertility has not yet been validated^9–11^. For further analysis of the WES data, variants were called and annotated as previously described^12–14^ using standard tools (Trimmomatic (https://www.plabipd.de/trimmomatic_main.html), BWA-MEM (Burrows-Wheeler Aligner: https://bio-bwa.sourceforge.net/), Picard (https://github.com/broadinstitute/picard) GATK (genomic analysis toolkit: https://gatk.broadinstitute.org/hc/en-us) and ANNOVAR (Annotate Variation: https://annovar.openbioinformatics.org/en/latest/) tools), and compared with data from fertile controls. Filtering was based on frequency (Minor allele frequency <1%), functional impact, testis-specific expression and relevance to spermatogenesis.

These analyses revealed two homozygous variants in the *MCMDC2* gene-(NM_173518.5): c.255_260del, and (NM_173518.5): c.260_261insGG-each with a frequency below 0.2% (gnomAD: https://gnomad.broadinstitute.org/). However, due to their adjacent positions, we interpret them as a single combined variant–(NM_173518.5): c.255_260delinsGG–validated by Sanger sequencing (Fig. 1A). The variants results in frameshift and early stop codon NM_173518.5 (NP_775789.3): p.Leu86ValfsTer12. This variant was not previously reported in gnomAD or other population databases. Segregation analysis could not be performed as parental samples were unavailable; therefore, parental genotypes were not obtained. The patient has seven half-brothers and sisters, but their fertility status is currently unknown. (Fig. 1B).

**Fig. 1:**
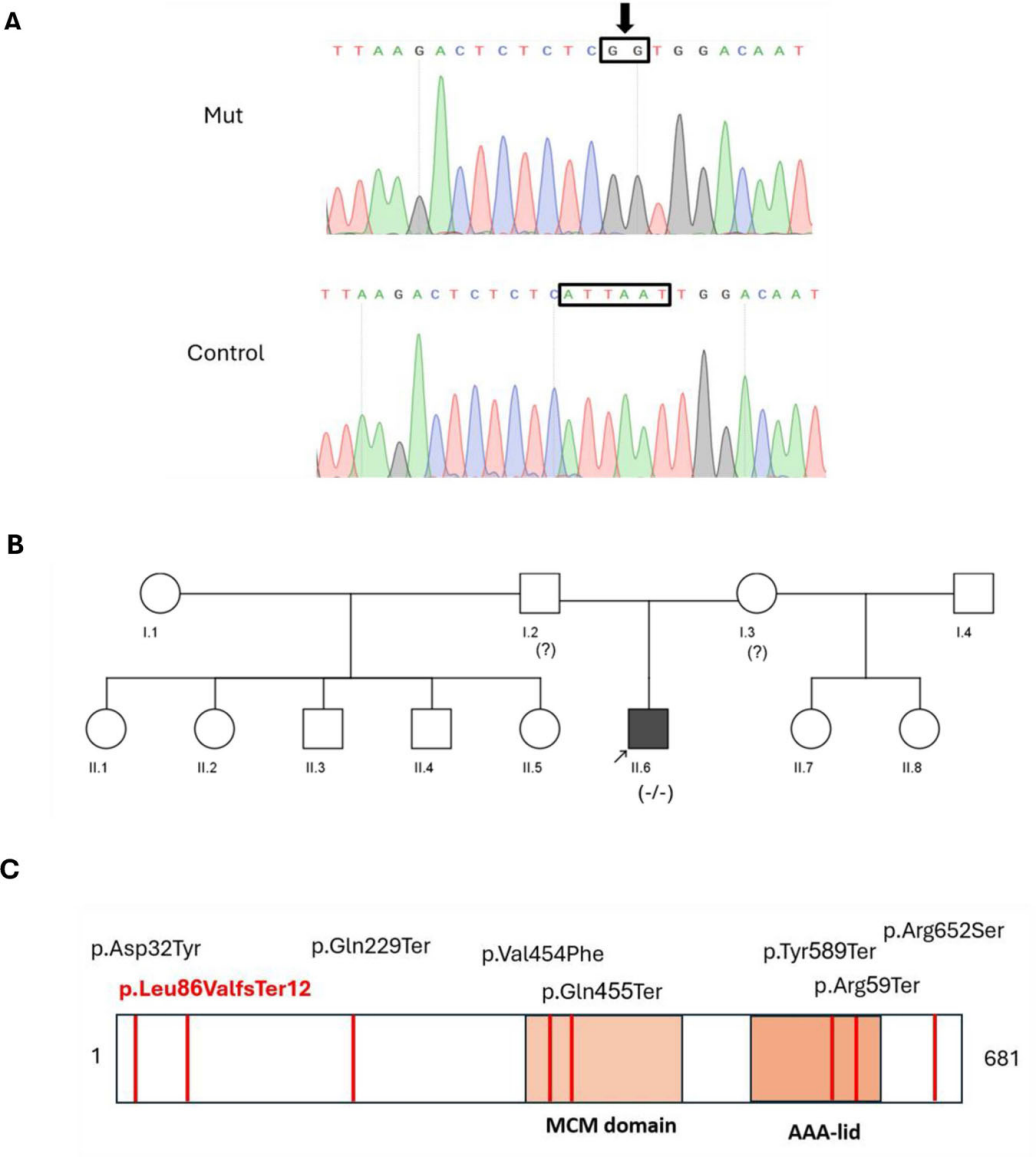
*MCMDC2* mutations. **A** The Sanger sequencing chromatogram in the proband showed a homozygous frameshift variant NM_173518.5: c.255_260delinsGG in the *MCMDC2* gene. **B** Family pedigree of the reported family generated using the CeGaT Pedigree Chart Designer. The genotype of the patient is homozygous mutated −/−, while the genotypes of the parents are unknown because they were not available for testing. **C** Scheme of the previously reported variants of the MCMDC2 protein with the variant observed in this case (Red).

To further evaluate the effect and distribution of the MCMDC2 p.Leu86ValfsTer12 variant, we screened our WES cohort of 113 men with impaired fertility, as well as additional WES data from 408 individuals from a mixed Israeli population, broadly representing the local population^15^. The variant was not detected in any individual within these two datasets. Notably, we identified the variant NM_173518.5: c.255_260del in heterozygosity in two brothers of Ashkenazi Jewish ancestry, unrelated to the studied patient, who were evaluated for infertility due to severe oligo-terato-asthenozoospermia and NOA. The variant was absent in their third brother (also affected with severe oligo-terato-asthenozoospermia) and in their fertile father. Importantly, because MCMDC2-associated azoospermia has so far been reported only in individuals carrying biallelic pathogenic variants (homozygous or compound heterozygous), and because the third affected brother did not carry the variant, the single heterozygous c.255_260del variant in these brothers is not considered causal for their infertility phenotype. Instead, this observation suggests that the variant may represent an ancestry-enriched carrier allele in the Ashkenazi population. This interpretation is supported by population stratification in gnomAD, where the variant shows its highest allele frequency among Ashkenazi Jews (minor allele frequency ~0.002), more than an order of magnitude higher than the global frequency (https://gnomad.broadinstitute.org/).

According to the American College of Medical Genetics and Genomics/Association for Molecular Pathology guidelines, the variant p.Leu86ValfsTer12 was classified as likely pathogenic: PVS1 was applied because it is a loss-of-function (LOF) variant, PM2 was applied because it is absent in the population databases, and PP4 was applied because the *MCMDC2* gene matches with the patient’s phenotype (NOA)^16^.

Additional variants in the *MCMDC2* gene, NM_173518.5 (NP_775789.3), have been reported in association with azoospermia in humans (Fig. 1C and Table 1). All of the variants were observed in a homozygous or compound heterozygous manner^11,17,18^. A comparison of the clinical information of all reported cases with MCMDC2 variants is presented in Table 1.

MCMDC2 is a nuclear protein implicated in DNA damage repair and meiotic recombination^19^. It is a distant member of the MCM protein family and contains two conserved domains: the MCM domain, which participates in the helicase complex responsible for DNA unwinding during replication initiation, and the AAA (ATPase) domain (Fig. 1C), which provides energy for the complex activity through ATP hydrolysis^16^. In addition, *MCMDC2* is highly expressed in the testis according to GTEx (https://www.gtexportal.org/home/). The frameshift variant detected in this study occurs at codon 86 in exon 2, an early position in the 15-exon transcript. Frameshift variants at such early sites typically introduce a premature termination codon that activates nonsense-mediated mRNA decay^20^. Thus, the principal mechanism of LOF is probably the degradation of the mutant transcript, resulting in markedly reduced gene expression. In the unlikely event that some mutant transcripts escape nonsense-mediated mRNA decay, any resulting truncated protein would be severely shortened and would lack the key MCM and AAA-ATPase domains, rendering it nonfunctional. Together, transcript degradation and loss of essential domains provide a strong mechanistic basis for a LOF effect. The proband of this family presented with NOA and histological evidence of maturation arrest. This pattern is consistent with the majority of previously reported patients harboring *MCMDC2* mutations, who demonstrated meiotic or spermatogenic arrest at the meiotic stage (Table 1). The hormonal profile of the proband of this family revealed normal FSH (6.1 IU/l) and LH (6.0 IU/l) levels, accompanied by low testosterone (8.5 nmol/l), indicating hypogonadism probably due to primary testicular failure. This endocrine profile aligns with other reported cases, where FSH levels ranged from normal (for example, 6.71 IU/l in M3) to markedly elevated (for example, 33.7 IU/l in participant no. 33), and testosterone levels varied from low to normal^11,17^ (Table 1). Testicular volumes in the proband of this family were bilaterally reduced (8–9 ml), falling at the lower end of the range compared with other reported cases. Taken together, although the proband of this family exhibits normal gonadotropin levels, the combination of small testicular volume and low testosterone supports a diagnosis of primary hypogonadism.

Notably, while most patients exhibited meiotic arrest, patient M1 demonstrated hypospermatogenesis-a finding we consider clinically atypical in the context of the cohort. This may be explained by a reduced impact of his missense variant (p.Asp32Tyr) on conformational changes in the protein structure and its interactions with surrounding residues, compared with the missense variant p.Arg652Ser within the functional domain of patient M3 and the LOF mutations of the other patients. Furthermore, in vitro analysis revealed that, unlike the other variants, the p.Asp32Tyr variant did not cause substantial changes in the MCMDC2 protein content or size. Because this variant is located at the end of an exon, additional computational splicing analysis was performed. SpliceAI (https://spliceailookup.broadinstitute.org/) predicted a loss of the original donor site with a decreased confidence score of 0.70, FruitFly suggested an effect on the normal splicing pattern, and Rare Disease Data Center Splice Change exhibited a decreased donor site score of 0.59, collectively supporting a substantial potential to disrupt normal splicing^17^.

In conclusion, the present findings support our hypothesis that the variant found in our patient is the main cause of his azoospermia.

## HGV Database

The relevant data from this Data Report are hosted at the Human Genome Variation Database at 10.6084/m9.figshare.hgv.3610. 10.6084/m9.figshare.hgv.3613. 10.6084/m9.figshare.hgv.3616. 10.6084/m9.figshare.hgv.3619. 10.6084/m9.figshare.hgv.3622. 10.6084/m9.figshare.hgv.3625. 10.6084/m9.figshare.hgv.3628. 10.6084/m9.figshare.hgv.3631. 10.6084/m9.figshare.hgv.3607.

## Supplementary information

Institutional review board approval doc
