# Supplementary material for: A novel loss-of-function mutation in MCMDC2 is associated with male infertility
Source: Hum Genome Var. 2026 Mar 12;13:5. doi: 10.1038/s41439-026-00342-6 (PMC12988226; doi:10.1038/s41439-026-00342-6)
Supplement: Supplementary file 1 — Institutional review board approval doc [file 41439_2026_342_MOESM1_ESM.pdf]

הנוהל לניסויים רפואיים בבני אדם 2020

טופס 7 א

אישור המנהל להארכת תוקף ביצוע הניסוי הרפואי

לכבוד תאריך: 14–אוקטובר–2024

החוקר הראשי: ד"ר נעמה שטיינר

מחלקה: נשים ויולדות א

סורוקה

הנדון: הארכת תוקף האישור לביצוע ניסוי רפואי בבני אדם

בהתאם לבקשתך, ניתן בזה אישור להמשך ביצוע הניסוי לפי המסמכים הבאים:

פרטי הניסוי

**מספר בקשה בוועדה מוסדית:** 0108-12-SOR	**סוג הניסוי:** גנטי
**מספר הבקשה במשרד הבריאות:**	**מספר MOH:**
**נושא הניסוי (בעברית):** Identification of the mutations causing male infertility	
**שם מוצר המחקר:**	**שם היזם:** ד"ר נעמה שטיינר<br>-

מסמכי הניסוי

מסמכי הניסוי	גרסה	תאריך
**פרוטוקול הניסוי- שם/מספר:**<br>1	3	21/07/2020
**טופס הסכמה- שם/מספר:**<br>קבוצת החולים במחלה הנחקרת עברית<br>קבוצת המשתתפים הנושאים את הגן הנחקר עברית<br>קבוצת הביקורת של משתתפים שאינם חולים במחלה הנחקרת עברית<br>קבוצת הביקורת של משתתפים שאינם חולים במחלה הנחקרת ערבית<br>קבוצת החולים במחלה הנחקרת ערבית<br>טופס הסכמה גנטיקה 2023 - גרסה 3 עברית<br>טופס הסבר על המחקר 2023 - גרסא 1 בריאים - נקי עברית<br>טופס הסבר על המחקר 2023 - גרסא 1 חולים - נקי עברית<br>טופס הסכמה ערבית - חולים ערבית<br>טופס הסכמה ערבית - בריאים ערבית	1.1<br>1<br>2<br>2.4<br>2.2<br>3<br>1<br>1<br>1<br>1	04/04/2012<br>21/07/2020<br>20/06/2022<br>27/11/2012<br>27/11/2012<br>04/06/2023<br>04/06/2023<br>04/06/2023<br>04/06/2023<br>04/06/2023
**חוברת לחוקר- שם/מספר:**		
**טופס 11 – מכתב לרופא המטפל**		

תנאים והגבלות לאחר אישור המחקר:

העברת דגימות השמורות במאגרי דגימות מאושרים, לגורמים שלישיים - חוקרים כמסחריים- תתבצע רק לאחר שנבחנה בקשת ההעברה על ידי ועדת הלסינקי המוסדית של בה"ח אשר לו אושרה הקמת המאגר וניתן אישורה לכך.
הניסוי מאושר ל- 100 משתתפים בלבד

אישור המנהל להארכת תוקף ביצוע הניסוי הרפואי

בתוקף ההסמכה שקיבלתי מהמנהל הכללי של משרד הבריאות, לתת אישור כ"מנהל" לעשיית ניסוי רפואי בבני-אדם, ולאחר שהבקשה להארכת תוקף אושרה על-ידי ועדת הלסינקי המוסדית מיום 14/10/2024 שוכנעתי כי הניסוי הרפואי הנו בהתאם לעקרונות של הצהרת הלסינקי ותקנות בריאות העם (ניסויים רפואיים בבני-אדם) תשמ"א-1980, וכי חוזה ההתקשרות בין היזם, החוקר הראשי והמוסד הרפואי עומד בדרישות הנוהל לניסויים רפואיים בבני אדם, הנני מאשר את ביצוע הניסוי בכפוף לתנאים הבאים:

תנאי האישור

1) הניסוי הרפואי יבוצע בכפוף להצהרת החוקר הראשי ו/או הרופא האחראי (טופס 1).

2) כל שינוי, תוספת או חריגה מפרוטוקול הניסוי הרפואי, טעון אישור בכתב של ועדת הלסינקי של המוסד הרפואי ו/או של משרד הבריאות.

3) הארכת תוקף האישור: **שלושה חודשים בטרם חלוף התקופה המאושרת לניסוי הרפואי**, חובה על החוקר הראשי להעביר דו"ח התקדמות על מהלך הניסוי לוועדת הלסינקי של המוסד הרפואי. הוועדה תודיע על החלטתה לגבי המשך הניסוי למנהל המוסד הרפואי. המנהל ינפיק אישור חדש לניסוי הרפואי.

4) בתום הניסוי יגיש החוקר הראשי, לוועדת הלסינקי דו"ח מסכם על מהלך הניסוי ותוצאותיו.

5) האישור ניתן לחוקר הראשי ולמוסד הרפואי המצוינים לעיל ואינו ניתן להעברה לאחר.

6) אספקת מוצר המחקר (IMP) למוסד הרפואי בו נערך הניסוי הרפואי היא באחריות יזם הניסוי. אחסונו וניפוקו של מוצר המחקר למטופלים הם באחריות החוקר הראשי. במקרים של תכשירים רפואיים, פעולות אלו יבוצעו באמצעות בית המרקחת המוסדי, אלא אם כן ועדת הלסינקי החליטה אחרת.

7) שמירת מסמכים: יש לשמור את כל מסמכי הבקשה, האישורים וכל המסמכים הנאספים במהלך הניסוי הרפואי **לפחות 15 שנים מתום הניסוי**.

8) שמירת הדגימות והמידע הגנטי: הדגימות והמידע הגנטי יישמרו לתקופה של **20 שנים מיום מתום הניסוי**. ניתן לבקש הארכת התקופה האמורה.

9) תוקף האישור: **27/12/2025**

ב ה צ ל ח ה !

בכבוד רב,

מנהל המרכז הרפואי
פרופ' שלומי קודש

פרופ' שלומי קודש
מ.ר 1-28855
מנהל/ת בית החולים
הנהלה
המרכז הרפואי האוניברסיטאי סורוקה

__העתק:__
יו"ר ועדת הלסינקי פרופ איתן לוננפלד
במקרה של ניסוי בתכשיר- מנהל בית המרקחת
היזם / נציגו בארץ
המחלקה לניסויים קליניים
